# Vitamin E intake is inversely associated with NAFLD measured by liver ultrasound transient elastography

**DOI:** 10.1038/s41598-024-52482-w

**Published:** 2024-01-31

**Authors:** Xiangjun Qi, Jiayun Guo, Yanlong Li, Caishan Fang, Jietao Lin, Xueqing Chen, Jie Jia

**Affiliations:** 1 The First Clinical School of Guangzhou University of Chinese Medicine, Guangzhou, 510405 China; 2 Hospital of Chengdu University of Traditional Chinese Medicine, Chengdu, 610000 China; 3 The First Affiliated Hospital of Guangzhou University of Chinese Medicine, No.12, Ji Chang Road, Baiyun District, Guangzhou, 510405 China

**Keywords:** Hepatitis, Liver diseases, Nutrition disorders, Biomarkers, Health care, Medical research, Risk factors

## Abstract

Non-alcoholic fatty liver disease (NAFLD) is one of the most common chronic liver diseases, whose severe form is associated with oxidative stress. Vitamin E as an antioxidant has a protective potential in NAFLD. Whether dietary intake of vitamin E, supplementary vitamin E use, and total vitamin E have a preventive effect on NAFLD requires investigation. A cross-sectional study used data from the National Health and Nutrition Examination Survey (2017–2020) was conducted. Vitamin E intake, including dietary vitamin E, supplementary vitamin E use, and total vitamin E, was obtained from the average of two 24-h dietary recall interviews. The extent of hepatic steatosis was measured by liver ultrasound transient elastography and presented as controlled attenuated parameter (CAP) scores. Participants were diagnosed with NAFLD based on CAP threshold values of 288 dB/m and 263 dB/m. The statistical software R and survey-weighted statistical models were used to examine the association between vitamin E intake and hepatic steatosis and NAFLD. Overall, 6122 participants were included for NAFLD analysis. After adjusting for age, gender, race, poverty level index, alcohol consumption, smoking status, vigorous recreational activity, body mass index, abdominal circumference, hyperlipidemia, hypertension, diabetes, and supplementary vitamin E use, dietary vitamin E was inversely associated with NAFLD. The corresponding odds ratios (OR) and 95% confidence intervals (CI) of NAFLD for dietary vitamin E intake as continuous and the highest quartile were 0.9592 (0.9340–0.9851, *P* = 0.0039) and 0.5983 (0.4136–0.8654, *P* = 0.0091) (*P*_trend_ = 0.0056). Supplementary vitamin E was significantly inversely associated with NAFLD (fully adjusted model: OR = 0.6565 95% CI 0.4569–0.9432, *P* = 0.0249). A marginal improvement in total vitamin E for NAFLD was identified. The ORs (95% CIs, *P*) for the total vitamin E intake as continuous and the highest quartile in the fully adjusted model were 0.9669 (0.9471–0.9871, *P* = 0.0029) and 0.6743 (0.4515–1.0071, *P* = 0.0538). Sensitivity analysis indicated these findings were robust. The protective effects of vitamin E significantly differed in the stratum of hyperlipidemia (*P*_interaction_ < 0.05). However, no statistically significant results were identified when the threshold value was set as 263 dB/m. Vitamin E intake, encompassing both dietary and supplemental forms, as well as total vitamin E intake, demonstrated a protective association with NAFLD. Augmenting dietary intake of vitamin E proves advantageous in the prevention of NAFLD, particularly among individuals devoid of hyperlipidemia.

## Introduction

Non-alcoholic fatty liver disease (NAFLD) is emerging as a major public health concern worldwide, with approximately 23–32% of the general population affected, and is the second-most frequent indication for liver transplantation^1–3^. NAFLD is characterized by hepatic steatosis, while progressive NAFLD is also accompanied by inflammation and hepatocyte ballooning, further leading to fibrosis, cirrhosis, and even malignant tumor^4^. Although the mechanisms underlying the progression of NAFLD remain unclear, previous studies have identified that oxidative stress plays an important role in NAFLD pathogenesis^5^, which made the administration of antioxidants a potentially effective strategy. A daily dietary intake of quality antioxidants could be recommended, but the association between dietary antioxidants and NAFLD has not been elucidated.

Vitamin E is a potent antioxidant, which can eliminate reactive oxygen species (ROS) and nitrogen species and increase the activity of antioxidative enzymes^6–8^. Thus, vitamin E has been extensively studied in NAFLD. Supplementary vitamin E has been found to improve non-alcoholic steatohepatitis (NASH) in randomized clinical trials^9,10^. Vitamin E supplementation was recommended in patients with proven NASH without diabetes by the American Association for the Study of Liver Disease^11^ and the European Association for the Study of the Liver^12^ guidelines. Since vitamin E is abundant in dairy foods, whether dietary vitamin E intake improves NAFLD remains a research hotspot.

Although a few studies have explored the association between dietary vitamin E and NAFLD^10,13,14^, there are some limitations in previous studies, such as small sample size, self-reported dietary habits rather than professionally assessed, lack of adjust for vitamin E supplementation and ignoring the position of total vitamin E intake. There is a need for further investigation due to the limitations of the study design and population in previous studies. Moreover, magnetic resonance imaging as well as serum biomarkers were used to diagnose NAFLD in the aforementioned studies^13,14^; however, liver ultrasound transient elastography is gradually becoming a more convenient and cost-effective examination, so it is more relevant to explore NAFLD diagnosed by liver ultrasound transient elastography. In this study, we examined the association of vitamin E intake with controlled attenuated parameter (CAP) score and NAFLD measured by liver ultrasound transient elastography in the US population, which may extend the application of vitamin E.

## Materials and methods

**Figure 1:**
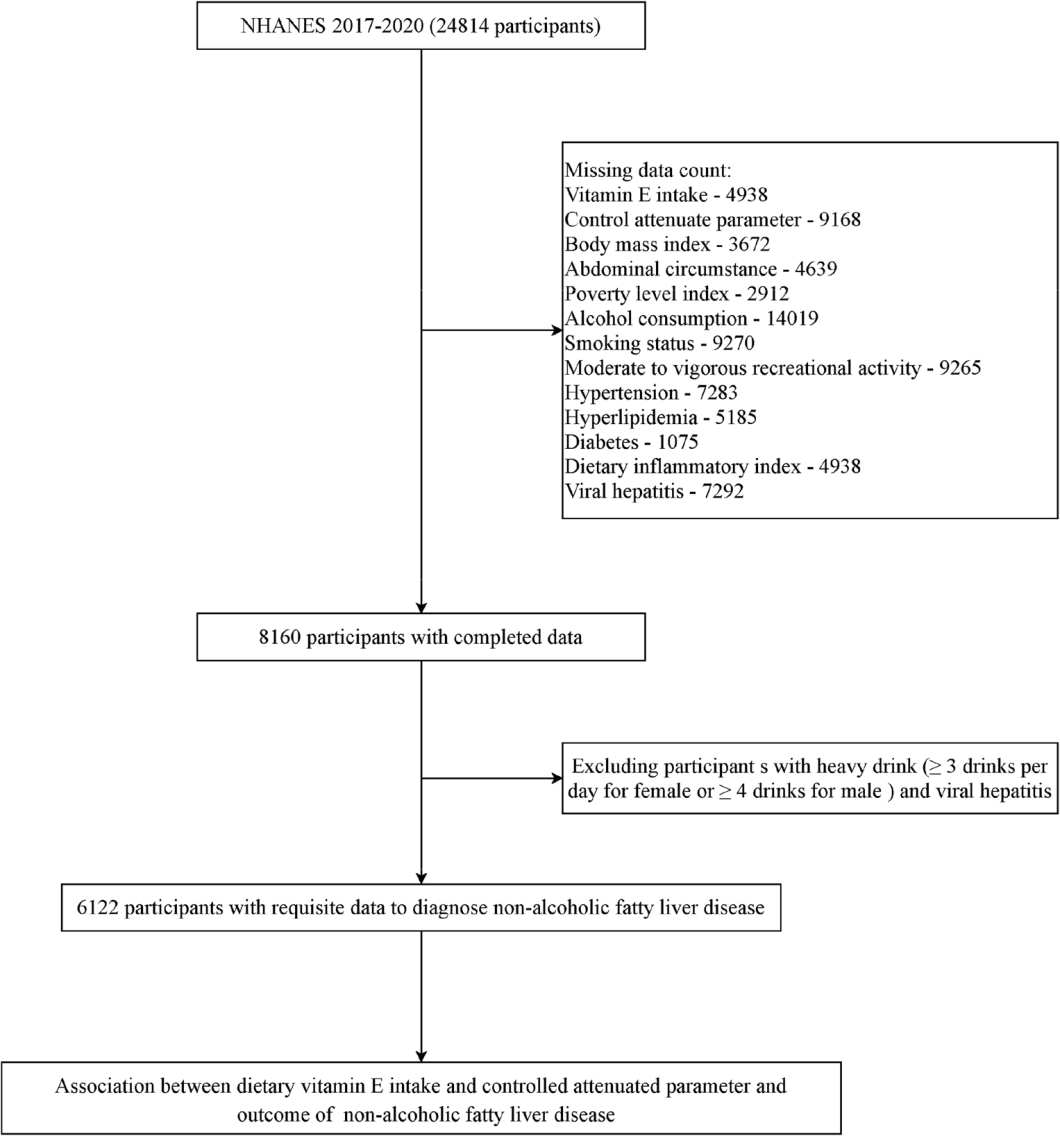
The overall design of the study and participants filtration. Abbreviation: NHANES, National Health and Nutrition Examination Survey.

The overall design of current study was shown in Fig. 1. We confirm that all methods were performed in accordance with the relevant guidelines and regulations for current research type.

### Participant

The National Health and Nutrition Examination Survey (NHANES) is conducted by the National Center for Health Statistics, a subdivision of the Centers for Disease Control and Prevention. The NHANES database provides demographic, socioeconomic, dietary, and health-related data for health assessment. NHANES became a continuous program in 1999, with approximately 5000 individuals from across the United States examined each year. Participants in the NHANES 2017–2020, who possessed whole dietary vitamin E intake data, supplementary vitamin E intake data and liver ultrasound transient elastography data were considered to be candidates for the current study.

Individuals with complete information about dietary vitamin E intake and CAP score measured by liver ultrasound transient elastography were included in the study, while those diagnosed with viral hepatitis were excluded. Figure 1 shows a flowchart of the excluded participants and the reasons for exclusion.

All NHANES study protocols were approved by the institutional review board of the National Center for Health Statistics. Oral and written informed consent were obtained from all participants^15^.

### Measurement of dietary vitamin E and supplementary vitamin E intake

The dietary intake data of NHANES participants was processed with the joint help of NHANES, the US Department of Agriculture (USDA), and the US Department of Health and Human Services. The participants underwent two 24-h dietary recall interviews through Mobile Examination Center and the duration between the two interviews was 3–10 days. The average daily intake of vitamin E (alpha-tocopherol) and supplementary vitamin E intake were calculated and used as the exposure factor. Total vitamin E intake was the sum of dietary vitamin E intake and supplementary vitamin E intake. Both the NHANES and USDA official websites provide information on how they conduct dietary recalls, serving as a reference for researchers^16–18^.

### Measurement of CAP and NAFLD

CAP score was measured by liver ultrasound transient elastography with the FibroScan 502 V2 Touch. The CAP score has been shown to be positively correlated with hepatic steatosis and it is also utilized as an indicator for NAFLD. Both CAP thresholds, 288 dB/m and 263 dB/m, were used for NAFLD diagnosis^19,20^. The eligible examined cases were required to meet the following criteria: fasting time ≥ 3 h, ≥ 10 complete stiffness (E) measures, and a liver stiffness interquartile range/median E < 30%.

### Collection of covariates

Based on previous literature, potential social, demographical, lifestyle and metabolism confounding factors were collected. Sociodemographic characteristics included age, gender (male and female), race (Mexican American, other Hispanic, non-Hispanic white, non-Hispanic black, and other), and poverty level index (≤ 1.3, 1.5–1.85, > 1.85). Lifestyle characteristics included alcohol consumption (never, mild, moderate, and heavy), smoking status (never, former, and current), and vigorous recreational activity (Yes and No). Never drinkers were ascertained by the questionnaire: “Ever had a drink of any kind of alcohol?” Furthermore, male participants who had ≥ 4 drinks per day, ≥ 3 drinks per day, and 0–3 drinks per day were classified as heavy, moderate, and mild drinkers, respectively. Female participants who had ≥ 3 drinks per day, ≥ 2 drinks per day, and 0–1 drinks per day were classified as heavy, moderate, and mild drinkers, respectively. Dietary inflammatory index was also calculated to evaluate the overall qulity of a participant’s diet^21^. Metabolic characteristics included body mass index (BMI), abdominal circumstances, hyperlipidemia, hypertension, and diabetes. Hyperlipidemia was defined by high-density lipoprotein (HDL) < 1.0 mmol/l in men, < 1.3 mmol/l in women or triglycerides ≥ 1.8 mmol/l regardless of gender. Hypertension was defined as systolic blood pressure ≥ 130 mmHg and/or diastolic blood pressure ≥ 80 mmHg on ≥ 3 occasions. Moreover, participants who answered “yes” to the questions: “Are you now taking prescribed medicine for high blood pressure?” and “Ever told you had high blood pressure?” were also defined as having hypertension. Diabetes was defined as a positive response to the question “Doctor told you have diabetes?”. Additionally, participants who achieved one or more of the following conditions were diagnosed with diabetes: glycohemoglobin ≥ 6.5%, fasting glucose ≥ 7 mmol/L, two-hour glucose of oral glucose tolerance test blood glucose ≥ 11.1 mmol/L, random serum glucose ≥ 11.1 mmol/L or antidiabetics use. Impaired fasting glucose was diagnosed with a two-hour glucose range of 6.1 mmol/L to 7.0 mmol/L on the oral glucose tolerance test.

### Statistical analysis

Survey-weighted statistical models were used for data analysis. Continuous variables were presented as mean ± standard error and categorical variables were expressed as frequencies and percentages. Weighted χ^2^ test (categorical variable), one-way ANOVA (normal distribution), or Kruskal–Wallis H test (skewed distribution) were employed to detect the differences between the low- and high- CAP score groups. Survey-weighted generalised linear models were used to test the association between vitamin E intake and CAP scores as well as NAFLD. A total of three statistical models were constructed in each analysis. Model I was the non-adjusted model with no covariates adjusted. Model II was the minimally adjusted model with only age and gender adjusted. Model III was the fully adjusted model with covariates presented in Table 1. We performed subgroup analyses between strata of hypertension, hyperlipidemia and diabetes. A p-value for interaction was used to determine whether the stratification effect was significant. Sensitivity analysis was conducted by excluding the participants who never drink.

**Table 1:** Baseline characteristics of participants by diagnoses of non-alcoholic fatty liver disease with threshold controlled attenuated parameter of 288 dB/m and 263 dB/m.

Variable	< 288 dB/m	≥ 288 dB/m	*P*-value	< 263 dB/m	≥ 263 dB/m	*P*-value
Vitamin E intake						
Dietary Vitamin E, mean(standard error), mg/d	9.88 (0.22)	8.94 (0.25)	0.003	9.97 (0.26)	9.11 (0.20)	0.004
Supplementary Vitamin E, n (%)			< 0.0001			< 0.0001
No	3483 (83.44)	1897 (90.57)		2703 (82.80)	2677 (89.24)	
Yes	523 (16.56)	219 (9.43)		423 (17.20)	319 (10.76)	
Total Vitamin E, mean(standard error), mg/d	11.06 (0.34)	9.47 (0.30)	< 0.001	11.20 (0.39)	9.76 (0.25)	< 0.001
Sociodemographic characteristic						
Age, mean(standard error), year	45.19 (0.69)	51.71 (0.56)	< 0.0001	43.78 (0.70)	51.43 (0.62)	< 0.0001
Gender, n (%)			< 0.0001			< 0.0001
Female	2287 (57.45)	954 (44.14)		1793 (57.76)	1448 (47.63)	
Male	1719 (42.55)	1162 (55.86)		1333 (42.24)	1548 (52.37)	
Race, n (%)			< 0.0001			< 0.0001
Non-Hispanic Black	1072 (11.64)	432 (8.34)		866 (11.83)	638 (9.08)	
Other Race—Including Multi-Racial	790 (10.10)	361 (8.38)		615 (9.62)	536 (9.43)	
Non-Hispanic White	1421 (66.34)	834 (68.45)		1115 (67.16)	1140 (66.90)	
Mexican American	364 (5.66)	307 (9.35)		252 (5.04)	419 (8.99)	
Other Hispanic	359 (6.25)	182 (5.48)		278 (6.35)	263 (5.60)	
Poverty level index, n (%)			0.93			0.06
< = 1.30	1102 (17.62)	563 (18.12)		871 (17.12)	794 (18.54)	
> 1.30, < = 1.85	583 (11.36)	327 (11.36)		440 (10.39)	470 (12.46)	
> 1.85	2321 (71.02)	1226 (70.51)		1815 (72.49)	1732 (69.00)	
Metabolic characteristic						
Abdominal circumference, mean(standard error), cm	93.17 (0.43)	112.74 (0.63)	< 0.0001	90.59 (0.58)	110.02 (0.57)	< 0.0001
Body mass index, mean(standard error), kg.m2	26.99 (0.18)	34.33 (0.30)	< 0.0001	26.04 (0.23)	33.29 (0.24)	< 0.0001
Hypertension, n (%)			< 0.0001			< 0.0001
No	2795 (76.18)	1057 (49.48)		2269 (79.20)	1583 (53.76)	
Yes	1211 (23.82)	1059 (50.52)		857 (20.80)	1413 (46.24)	
Hyperlipidemia, n (%)			< 0.0001			< 0.0001
No	1628 (41.63)	390 (17.09)		1402 (45.57)	616 (19.69)	
Yes	2378 (58.37)	1726 (82.91)		1724 (54.43)	2380 (80.31)	
Diabetes, n (%)			< 0.0001			< 0.0001
No	3334 (87.33)	1232 (60.35)		2701 (90.13)	1865 (64.96)	
Impaired fasting glucose	235 (6.30)	183 (9.35)		153 (5.46)	265 (9.43)	
Yes	437 (6.37)	701 (30.29)		272 (4.42)	866 (25.60)	
Lifestyle characteristic						
Smoking status, n (%)			0.01			0.01
Former	790 (22.62)	569 (27.92)		581 (21.99)	778 (27.11)	
Never	2758 (67.74)	1317 (63.76)		2161 (68.08)	1914 (64.52)	
Now	458 (9.64)	230 (8.32)		384 (9.93)	304 (8.37)	
Dietary inflammatory index, mean(standard error)	1.40 (0.07)	1.54 (0.08)	0.16	1.37 (0.08)	1.53 (0.07)	0.06
Moderate to vigorous recreational activity, n (%)			< 0.0001			< 0.0001
No	971 (18.42)	632 (25.47)		706 (17.10)	897 (24.93)	
Yes	3035 (81.58)	1484 (74.53)		2420 (82.90)	2099 (75.07)	
Alcohol consumption, n (%)			0.03			0.1
Never	617 (10.09)	319 (12.69)		498 (10.79)	438 (11.15)	
Mild	2230 (58.45)	1292 (61.66)		1734 (57.75)	1788 (61.53)	
Moderate	1159 (31.45)	505 (25.65)		894 (31.46)	770 (27.32)	

Data analysis was performed with the statistical software packages R (http://www.R-project.org). Survey-weighted statistical models were constructed with the *survey* package of R. All statistical tests were two-sided, and a *P* value < 0.05 was considered as statistically significant.

### Ethics statement

The current study was supported by the Ethics Review Board of U.S. National Center for Health Statistics, and written informed consents were obtained from all participants of the NAHNES survey.

## Results

Table 1 presents the baseline sociodemographic, lifestyle, and metabolic characteristics of all the participants based on two CAP thresholds. Participants with CAP scores greater than the thresholds tended to have the following characteristics: lower intake of vitamin E, less use of supplemental vitamin E, older, a higher proportion of males, higher level of BMI, higher rate of smoking history, hyperlipidemia, hypertension, and diabetes, but a lower proportion of moderate to vigorous recreational activity.

**Figure 2:**
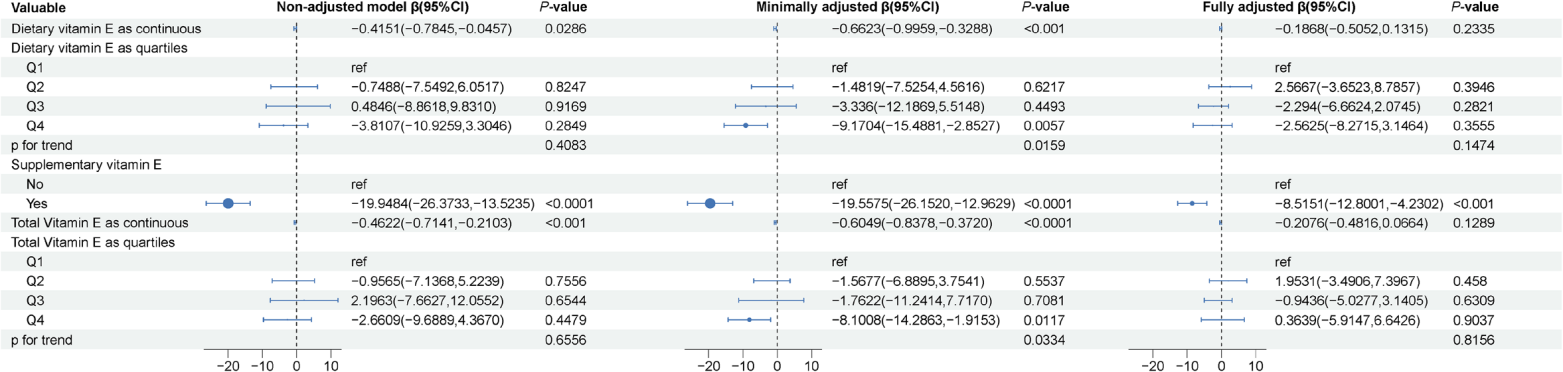
Weighted association between vitamin E intake and controlled attenuated parameter. Non-adjusted model: no covariates were adjusted. Minimally adjusted model: age and gender were adjusted. Fully adjusted model: age, gender, race, poverty level index, alcohol consumption, smoking status, moderate to vigorous recreational activity, body mass index, abdominal circumference, hypertension, diabetes and hyperlipidemia were adjusted. Supplementary vitamin E was also adjusted when exposure was to dietary vitamin E and total vitamin E. Abbreviation: CI, confidence interval.

The association between vitamin E intake and CAP score was demonstrated in the three models and the corresponding effect size (β) with 95% confidence intervals (CIs) is provided in Fig. 2. In the non-adjusted model, dietary vitamin E intake showed a negative correlation with CAP score (β = − 0.4151, 95% CI − 0.7845 to − 0.0457, *P* = 0.0286). In the minimally adjusted model, dietary vitamin E intake presented a significant negative correlation with CAP score (β = − 0.6623, 95% CI − 0.9959 to − 0.3288, *P* < 0.001). However, we did not find statistically significant differences in the results in the fully adjusted model. Results for dietary vitamin E intake as quartiles were not significant. Although supplemental vitamin E use was significantly negatively associated with CAP scores (β = − 8.5151, 95% CI − 12.8001 to − 4.2302, *P* < 0.001), we did not obtain a positive result from the fully adjusted model of total vitamin E intake (β = − 0.2076, 95% CI − 0.4816 to 0.0664, *P* = 0.1289).

**Figure 3:**
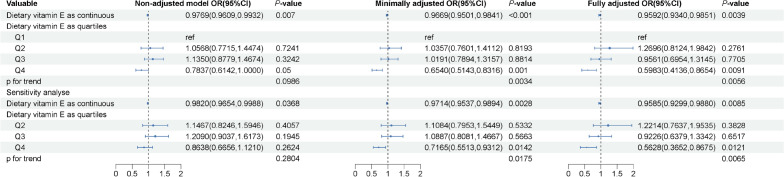
Weighted association between dietary vitamin E intake and non-alcoholic fatty liver disease at controlled attenuated parameter 288 dB/m. Non-adjusted model: no covariates were adjusted. Minimally adjusted model: age and gender were adjusted. Fully adjusted model: age, gender, race, poverty level index, alcohol consumption, smoking status, moderate to vigorous recreational activity, body mass index, abdominal circumference, hypertension, diabetes, hyperlipidemia and supplementary vitamin E were adjusted. Sensitivity analysis was implemented by excluding patients who never drank. Abbreviation: OR, odds ratio; CI, confidence interval.

**Figure 4:**
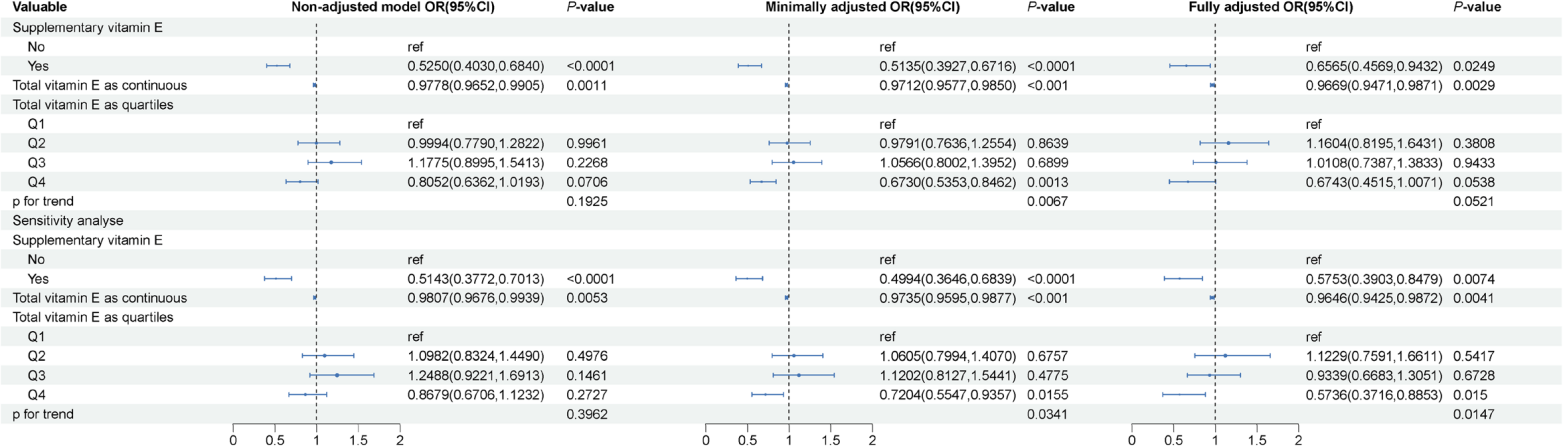
Weighted association between total vitamin E intake and non-alcoholic fatty liver disease diagnosed at controlled attenuated parameter 288 dB/m. Non-adjusted model: no covariates were adjusted. Minimally adjusted model: age and gender were adjusted. Fully adjusted model: age, gender, race, poverty level index, alcohol consumption, smoking status, moderate to vigorous recreational activity, body mass index, abdominal circumference, hypertension, diabetes and hyperlipidemia were adjusted. Supplementary vitamin E was also adjusted when exposure was to total dietary vitamin E. Sensitivity analysis was implemented by excluding patients who never drank. Abbreviation: OR, odds ratio; CI, confidence interval.

After removing the participants with heavy drinking and missing drinking data, a total of 2116 participants were diagnosed with NAFLD according to the threshold value of 288 dB/m. Figure 3 shows the odds ratios (OR) for dietary vitamin E intake in relation to NAFLD. The ORs (95% CIs, *P*) for the dietary vitamin E intake as continuous and the highest quartile in the fully adjusted model were 0.9592 (0.9340–0.9851, *P* = 0.0039) and 0.5983 (0.4136–0.8654, *P* = 0.0091). A statistically significant *P*_trend_ of 0.0056 was also identified. Sensitivity analyses yielded consistent positive results (0.9585 (0.9299–0.9880, *P* = 0.0085) and 0.5628 (0.3652–0.8675, *P* = 0.0121), *P*_trend_ = 0.0065). Figure 4 shows the ORs for total vitamin E intake in relation to NAFLD. Firstly, a protective effect of supplementary vitamin E use was identified, the corresponding OR (95% CI, *P*) is 0.6565 (0.4569–0.9432, *P* = 0.0249). The ORs (95% CIs, *P*) for the total vitamin E intake as continuous and the highest quartile in the fully adjusted model were 0.9669 (0.9471–0.9871, *P* = 0.0029) and 0.6743 (0.4515–1.0071, *P* = 0.0538). A marginally statistically significant *P*_trend_ of 0.0521 was specified. The protective effect of using supplemental vitamins still stands in the sensitivity analysis (OR = 0.5753, 95% CI 0.3903–0.8479, *P* = 0.0074). The association between total vitamin E intake and NAFLD was more significant in the sensitivity analysis. The ORs (95% CIs, *P*) in the sensitivity analysis for the total vitamin E intake as continuous and the highest quartile in the fully adjusted model were 0.9646 (0.9425–0.9872, *P* = 0.0041) and 0.5736 (0.3716–0.8853, *P* = 0.015). A statistically significant *P*_trend_ of 0.0147 was specified. However, when the diagnostic threshold was set at 263, the relationship between dietary vitamin intake and total vitamin E intake and NAFLD was not statistically significant (Supplementary Table 1).

Subgroup analysis for hypertension, hyperlipidemia and diabetes was presented in Table 2. The association between dietary vitamin E intake and NAFLD outcome did not differ between the stratum of hypertension (*P*_interaction_ = 0.748) and marginally differ between the stratum of diabetes (*P*_interaction_ = 0.088). Nevertheless, the association between dietary vitamin E intake and NAFLD outcome significantly differ between participants with hyperlipidemia (OR = 0.968, 95% CI 0.942–0.995, *P* = 0.022) or without hyperlipidemia (OR = 0.884, 95% CI 0.843–0.927, *P* < 0.0001) (*P*_interaction_ < 0.0001). The results of the subgroup analysis of total vitamin E intake were similar to those of dietary vitamin E intake, a statistically significant *P*_interaction_ of 0.027 was identified in the stratum of hyperlipidemia. No meaningful subgroup was found when the threshold for NAFLD was 263 dB/m (Supplementary Table 2).

**Table 2:** Interaction effects of hypertension, hyperlipidemia, and diabetes in the association between dietary vitamin E intake, total vitamin E intake and non-alcoholic fatty liver disease (288 dB/m).

	Dietary vitamin E			Total vitamin E		
Stratum	OR (95% CI)	*P*-value	p for interaction	OR (95% CI)	*P*-value	p for interaction
Hypertension			0.748			0.286
No	0.967 (0.937,0.998)	0.036		0.979 (0.960,0.998)	0.034	
Yes	0.941 (0.907,0.975)	0.002		0.946 (0.919,0.973)	< 0.001	
Hyperlipidemia			< 0.001			0.027
No	0.884 (0.843, 0.927)	< 0.0001		0.937 (0.896, 0.981)	0.007	
Yes	0.968 (0.942,0.995)	0.022		0.970 (0.952,0.989)	0.003	
Diabetes			0.088			0.088
No	0.951 (0.922,0.981)	0.003		0.965 (0.946,0.985)	0.001	
Impaired fasting glucose	0.967 (0.891, 1.049)	0.401		0.975 (0.918, 1.035)	0.382	
Yes	0.968 (0.914,1.024)	0.245		0.956 (0.916,0.997)	0.039	

OR, odds ratio; CI, confidence interval.

## Discussion

Although supplementary vitamin E intake has shown improvements as a randomized intervention in the treatment of non-alcoholic steatohepatitis, which is a severe form of NAFLD^9,10,22^, only a few cross-sectional studies focused on dietary vitamin E intake^14^. The present study analyzed the association between vitamin E intake including dietary vitamin E, supplemental vitamin E use, and total vitamin E intake, and the CAP measured by liver ultrasound transient elastography as well as the outcome of NAFLD in the US population. Participants with CAP score ≥ 288 dB/m had lower dietary and total intake of vitamin E. Dietary and total vitamin E intake was inversely associated with NAFLD outcome in the fully adjusted models. When stratified by hypertension, dyslipidemia and diabetes, significant interaction was detected in the stratum of hyperlipidemia. Sensitivity analysis indicated that the inverse association between vitamin E and NAFLD outcome was stable.

Randomized clinical trials have been conducted to demonstrate the role of vitamin E in the treatment of NASH. Significantly decreased hepatocellular ballooning point was observed in biopsy-diagnosed NASH patients who were administrated vitamin E (800 IU per day)^9^. However, no improvement in NASH by vitamin E (800 IU per day) intake was identified in the non-diabetic subgroup^10^. In a phenome-wide association study, dietary vitamin E demonstrated a negative association with NAFLD, but this protective effect was not significant in individuals with a BMI less than 25 kg/m^2^.^14^. These evidence suggests that vitamin E intake varies across population subgroups, and it makes sense to continue to explore potentially advantaged populations. As our study results indicate, there is an association between hyperlipidemia and the protective effect of vitamin E. Furthermore, several cross-sectional studies have focused on the relationship between dietary vitamin E intake and the NAFLD subgroup. A small sample cross-sectional study with 789 participants on an Israeli population aged 40–70 years showed that dietary intake of vitamin E reduced the risk of abdominal ultrasonography diagnosis of NAFLD^13^. Weiwen Chai et al.^23^ found that dietary vitamin E intake was inversely associated with liver ultrasound-diagnosed hepatic steatosis in NHANES 2017–2018, and the participants were diagnosed with steatosis based on a CAP threshold of 302 dB/m. Both studies had relatively small sample sizes, and in the second study, while an elevated CAP diagnostic threshold can enhance diagnostic accuracy, it concurrently reduces the sensitivity of diagnostic rates.

Vitamin E is a powerful antioxidant agent that is thought to be therapeutic in NAFLD. In vivo and in vitro experiments have been conducted to demonstrate its potential mechanisms. Progressive and severe NAFLD is accompanied by oxidative stress, mainly involving ROS production, endoplasmic reticulum (ES), iron accumulation, and transcription factor Nrf2^24^. Vitamin E prevented the progression of hepatic steatosis in phosphatidylethanolamine N-methyltransferase-deficient and high-fat diet mouse models, and the potential mechanisms included suppression of ER stress and hepatic ROS production^25^. Nrf2/CES1 signaling pathway, which regulates the generation of antioxidant molecules and further restrains oxidative stress, was activated by vitamin E^26^. However, other studies have reported conflicting results. Alcala et al.^27^ reported that short-term vitamin E intake impaired the ROS signaling and caused fatty liver in obese mice. This phenomenon may be explained by the differences in animal models, disease progression, and the duration or dosage of vitamin E administered. Hence, further studies are expected to investigate the biological mechanism underlying the association between dietary vitamin E intake and NAFLD.

This study had several advantages. The inverse association between vitamin E and NAFLD was expanded to the US adults. The study not only identified the association between dietary vitamin E and NAFLD outcomes but also revealed relationships between supplemental vitamin E use, total vitamin E, and NAFLD. Hypertension and hyperlipidemia are risk factors for NAFLD^28^. Previous studies did not explore variations in vitamin E intake within these two stratifications. Our research indicates that hyperlipidemia is a factor influencing the efficacy of vitamin E, as both dietary vitamin E and total vitamin E exhibit weakened protective effects in individuals with hyperlipidemia. Nevertheless, the study had some limitations.

A cross-sectional study design is incapable of determining the causality, therefore prospective longitudinal studies are expected in the future. Although two 24-h dietary recalls have been used by researchers in cross-sectional studies^29^, it does not fully reflect the dietary status of the participants, which may influence our assessment of exposure to some extent. While transient elastography is a widely employed non-invasive method for assessing liver steatosis, its effectiveness may be hindered by participant adiposity, the existence of perihepatic ascites, and restricted options for selecting an appropriate sampling area. When total vitamin E intake is treated as a categorical variable, the *P*-value and *P*_trend_ for the association between the highest quantile of total vitamin E intake and NAFLD is 0.0538 and 0.0521, respectively, while the results in the sensitivity analysis exhibit significant statistical differences, differing from dietary vitamin E. These findings suggest potential inadequacies in our study in two aspects. Firstly, the issue of sample size may contribute to result fluctuations, emphasizing the potential value of larger-scale cross-sectional studies. Secondly, insufficient adjustment for covariates related to supplemental vitamin E intake or NAFLD^30,31^ may be a contributing factor.

## Conclusion

Vitamin E intake was inversely associated with the NAFLD outcome measured by liver ultrasound transient elastography. Increasing dietary sources of vitamin E is beneficial for preventing NAFLD, particularly in individuals without hyperlipidemia.

### Supplementary Information

Supplementary Tables.

## Data Availability

The datasets used and/or analyzed in the current study are available in the article or supplementary material.
