# Supplementary material for: Vitamin E intake is inversely associated with NAFLD measured by liver ultrasound transient elastography
Source: Sci Rep. 2024 Jan 31;14:2592. doi: 10.1038/s41598-024-52482-w (PMC10831069; doi:10.1038/s41598-024-52482-w)
Supplement: Supplementary file 1 — Supplementary Tables. [file 41598_2024_52482_MOESM1_ESM.docx]

**Supplementary Table 1.** Weighted association between vitamin E intake and non-alcoholic fatty liver disease diagnosed at controlled attenuated parameter 263 dB/m.

|  | Non-adjusted model | | Minimally adjusted model | | Fully adjusted model | |
| --- | --- | --- | --- | --- | --- | --- |
|  | OR (95% CI) | *P*-value | OR (95% CI) | *P*-value | OR (95% CI) | *P*-value |
| Dietary vitamin E as continuous | 0.9797(0.9661,0.9935) | 0.0052 | 0.9714(0.9570,0.9861) | <0.001 | 0.9749(0.9539,0.9964) | 0.0247 |
| Dietary vitamin E as quartiles |  |  |  |  |  |  |
| Q1 | ref |  | ref |  | ref |  |
| Q2 | 1.0502(0.8436,1.3074) | 0.6533 | 1.0314(0.8327,1.2775) | 0.7710 | 1.2317(0.8943,1.6965) | 0.1883 |
| Q3 | 1.0393(0.8247,1.3096) | 0.7377 | 0.9352(0.7429,1.1772) | 0.5581 | 0.9099(0.6697,1.2364) | 0.5259 |
| Q4 | 0.8176(0.6678,1.0011) | 0.0512 | 0.6968(0.5645,0.8602) | 0.0014 | 0.7599(0.4715,1.2248) | 0.2425 |
| p for trend |  | 0.0746 |  | 0.0033 |  | 0.143 |
| Supplementary vitamin E |  |  |  |  |  |  |
|  | ref |  | ref |  | ref |  |
|  | 0.5805(0.4743,0.7104) | <0.0001 | 0.5619(0.4494,0.7024) | <0.0001 | 0.8000(0.6310,1.0143) | 0.0640 |
| Total vitamin E as continuous | 0.9814(0.9713,0.9916) | <0.001 | 0.9762(0.9655,0.9870) | <0.0001 | 0.9791(0.9635,0.9950) | 0.0128 |
| Total vitamin E as quartiles |  |  |  |  |  |  |
| Q1 | ref |  | ref |  | ref |  |
| Q2 | 1.0391(0.8408,1.2842) | 0.7159 | 1.0258(0.8324,1.2642) | 0.8056 | 1.2154(0.9055,1.6315) | 0.1808 |
| Q3 | 1.0902(0.8492,1.3996) | 0.4879 | 0.9821(0.7571,1.2740) | 0.8890 | 0.9859(0.7410,1.3119) | 0.9182 |
| Q4 | 0.8769(0.7189,1.0698) | 0.1888 | 0.7492(0.6116,0.9177) | 0.0066 | 0.9594(0.5806,1.5854) | 0.8644 |
| p for trend |  | 0.2698 |  | 0.0148 |  | 0.6565 |

**Non-adjusted model:** no covariates were adjusted.

**Minimally adjusted model:** age and gender were adjusted.

**Fully adjusted model:** age, gender, race, poverty level index, alcohol consumption, smoking status, moderate to vigorous recreational activity, body mass index, abdominal circumference, hypertension, diabetes and hyperlipidemia were adjusted. Supplementary vitamin E was also adjusted when exposure was to dietary vitamin E and total vitamin E.

**Abbreviation:** OR, odds ratio; CI, confidence interval.

**Supplementary Table 2.** Interaction effects of hypertension, hyperlipidemia, and diabetes in the association between dietary vitamin E intake, total vitamin E intake and non-alcoholic fatty liver disease (263 dB/m).

|  | Dietary vitamin E | | | Total vitamin E | | |
| --- | --- | --- | --- | --- | --- | --- |
| Stratum | OR(95% CI) | *P*-value | p for interaction | OR(95% CI) | *P*-value | p for interaction |
| Hypertension |  |  | 0.587 |  |  | 0.935 |
| No | 0.978(0.957,1.000) | 0.047 |  | 0.984(0.970,0.999) | 0.038 |  |
| Yes | 0.964(0.931,0.998) | 0.04 |  | 0.971(0.948,0.995) | 0.018 |  |
| Hyperlipidemia |  |  | 0.463 |  |  | 0.654 |
| No | 0.962(0.930,0.994) | 0.024 |  | 0.978(0.959,0.998) | 0.033 |  |
| Yes | 0.978(0.954,1.002) | 0.069 |  | 0.981(0.965,0.997) | 0.022 |  |
| DM |  |  | 0.528 |  |  | 0.667 |
| No | 0.970(0.946,0.994) | 0.015 |  | 0.977(0.962,0.993) | 0.006 |  |
| Impaired fasting glucose | 0.924(0.870,0.982) | 0.013 |  | 0.943(0.895,0.994) | 0.032 |  |
| Yes | 0.999(0.951, 1.049) | 0.96 |  | 0.987(0.942, 1.034) | 0.564 |  |

**Abbreviation:** OR, odds ratio; CI, confidence interval.
